# Spectral Properties of Highly Emissive Derivative of Coumarin with *N,N*-Diethylamino, Nitrile and Tiophenecarbonyl Moieties in Water-Methanol Mixture

**DOI:** 10.1007/s10895-019-02446-5

**Published:** 2019-11-21

**Authors:** Anna Kolbus, Andrzej Danel, Danuta Grabka, Mateusz Kucharek, Karol Szary

**Affiliations:** 1 Institute of Chemistry, The Jan Kochanowski University, Swietokrzyska 15G, 25-406 Kielce, Poland; 2 Institute of Chemistry, Faculty of Food Technology, University of Agriculture, Balicka St. 122, 31-149 Kraków, Poland; 3 Institute of Physics, The Jan Kochanowski University, Swietokrzyska 15G, 25-406 Kielce, Poland

**Keywords:** Derivative of coumarin, Binary solvent mixture, Quantum yield, Fluorescence study

## Abstract

The new derivative of coumarin (*E*)-3-[7-(diethyloamino)-2-oxo-chromen-3yl]-2-(tiophene-2-carbonyl)prop-2-enenitrile (NOSQ) was easy synthesized with commercial substrates as a result of the search of new Michael type addition sensors based on coumarins. Spectral properties of highly emissive NOSQ were investigated by steady state analysis (absorption and fluorescence measurements) and time-resolved analysis (fluorescence lifetime measurements). The effect of water-methanol mixture on the photophysical properties of the NOSQ molecule was analyzed. With increasing of volumetric fraction of water the intensity of absorbance and fluorescence was strongly reduced. The NOSQ quantum yield in methanol was quite high and the first portions of water caused a significant increase in this value. Water, which is usually a quencher, in this case caused the increase in the quantum yield. The fluorescence lifetimes had second-order decay and the values of fluorescence lifetime increased with increasing alcohol content. Density functional theory (DFT) calculations and experimental data remained in agreement and showed that the interaction between the NOSQ molecule and the solvent affects the appearance of the new conformer.

## Introduction

2*H*-Chromen-2-one commonly known as coumarin is an important natural oxygen heterocycle which was isolated for the first time from *tonka* seeds in 1820 [1, 2]. Forty eight years later an English chemist William Henry Perkin synthesized coumarin from salicylic aldehyde and acetic anhydride [3]. Without doubt neither the discoverers nor Perkin didn’t predicted that today derivatives of coumarin will find such numerous applications. Some of them are biologically active substances like naturally occurring anticoagulant substance- dicumarol [4]. Similar in mode of action is warfarin – initially a rat poison and at present an important medication [5]. Due to specific scent coumarin is used in tobacco and perfumery industry. Many coumarin derivatives exhibit strong emission with high fluorescence quantum yield and a superior photostability. Their photophysical properties can be easily modified by introduction of electro donating/accepting groups like NR_2_, OH, CN or by fusion with other aromatic groups leading to so-called π-expanded coumarins [6]. These compounds are widely used as laser dyes, optical brighteners or biological markers [7–9]. Coumarins were applied as emissive materials in organic light emitting diodes OLED too. For example Kodak patented coumarin based laser dye C-545 TB and used it as a dopant in a device ITO/NPB/AlQ_3_:1%C-545 TB/AlQ_3_/Mg:Ag. The device showed a saturated green emission with a luminescent efficiency of 12.9 Cd/m^2^, EQE of 3.68% and an impressive brightness of 64,991 cd/m^2^ [10]. Just recently Zheng et al. used two coumarin derivatives as thermally activated delayed fluorescence (TADF) emitters achieving the highest external quantum efficiency as far as coumarins is concerned so far. The values are 17.8% and 19.6% respectively with luminance reaching 10,000 cd/m^2^ [11]. Regardless of laser dyes or OLED luminophores in the recent years a significant growth in application of 2*H*-chromen-2-one derivatives as fluorescent chemosensors can be observed. Thus Yang and co-workers synthesized malononitrile coumarin derivative for HSO${}_3^{-}$ detection. In the presence of this ion a hypsochromic shift both in absorbance and emission is observed [12]. Biologically important thiols derivatives like cysteine, homocysteine and glutathione are important targets for fluorescent sensors. Quite a lot of them were prepared from 7-*N,N*-dialkyl-3-formylcoumarine via Knoevenagel condensation with activated methylene group *ex*. diethylmalonate or methylaryl ketones. The resulted *α,β*-unsaturated compounds exhibit usually weak fluorescence or doesn’t emit the light at all. After Michael addition of thiol type compound the fluorescence is recovered [13–15]. New analytical techniques of cations detecting are of great interest to scientists engaged in clinical biochemistry or natural environment protection. Derivatives of comarine can be used as the sensors of cations, e.g. Hg^2+^ where as a mercury receptor 1,4,7,10-tetraoxa-13-azacyclopentadecane was applied [16] or K^+^ for which 6,7-(4-methyl)coumaro[2,2,2]cryptand was designed for measuring potassium ions [17]. We synthesized the new derivative of coumarin: (*E*)-3-[7-(diethyloamino)-2-oxo-chromen-3yl]-2-(tiophene-2-carbonyl)prop-2-enenitrile (NOSQ) – promising candidate for Michael type addition sensor. In this paper we present the photophysical properties of NOSQ in methanol and in binary methanol – water mixture in consideration the possibility of later application in biological systems.

## Experimental

### Chemicals

(*E*)-3-[7-(diethyloamino)-2-oxo-chromen-3yl]-2-(tiophene-2-carbonyl)prop-2-enenitrile (denoted as NOSQ) was synthesized in the Institute of Chemistry at the Agricultural University in Krakow. Products used in the synthesis were received from POCH S.A.. Methanol had the spectroscopic purity and it was used as received from POCH S.A.

For spectroscopic measurements, solutions of NOSQ in organic solvent (methanol) and in binary mixture (water-methanol) were prepared. The concentration of NOSQ in pure methanol and all binary solvents was equal 1.06 × 10^−5^ M. The participation of volume of water and methanol in binary mixtures shows Table 1. It was considered mixtures containing no more than 40% of the volume of water in binary mixtures. Higher content of water caused precipitation of black sediment.

**Table 1:** Content of water and methanol in binary solvent mixtures

l.p.	Volume of water [cm^3^]	Volume of methanol [cm^3^]	Water content [% of volume]
1.	0	2.5	0
2.	0.1	2.4	4
3.	0.2	2.3	8
4.	0.3	2.2	12
5.	0.4	2.1	16
6.	0.5	2.0	20
7.	0.6	1.9	24
8.	0.7	1.8	28
9.	0.8	1.7	32
10.	0.9	1.6	36
11.	1	1.5	40

### Optical Properties Measurements

Measurements of absorption spectra were taken on the UV-3600 Shimadzu Spectrophotometr. Fluorescence spectra were recorded on the Hitachi F-7000 Spectrofluorymetr and calibrated on photomultiplayer sensivity using *N*,*N*-dimethylamino-m-nitrobenzene (C_8_H_10_N_2_O_2_), dissolved in mixture of *n*-hexane and benzene (proportions by volume respectively 70% *n*-hexane and 30% benzene). All spectra were carried out at room temperature. The excitation wavelength of the fluorescence was fixed as 510 nm. The fluorescence quantum yield, *ϕ*_*f*_, was calculated using 4-dimethyloamino-4-nitrostilbene in *o*-dichlorobenzene as the standard. The quantum yield of standard was taken equal 0.7 [18]. The refractive index, *n*, and the dielectric permittivity, *ε*, was calculated with respect to water and methanol participation:

1 $$ \mathrm{n}=\%{V}_{MeOH}\ast {\mathrm{n}}_{MeOH}+\%{V}_{H_2O}\ast {\mathrm{n}}_{H_2O} $$

2 $$ \upvarepsilon =\%{V}_{MeOH}\ast {\upvarepsilon}_{MeOH}+\%{V}_{H_2O}\ast {\upvarepsilon}_{H_2O} $$

The solvent polarity was calculated according to the equation [19]:

3 $$ f\left(\varepsilon, n\right)=\frac{\varepsilon -1}{\varepsilon +2}-\frac{1}{2}\frac{n^2-1}{n^2+2} $$

The fluorescence lifetime was measured using the Pico Quant compact FLIM and FCS module based on Nikon A1R confocal microscope. The solution was transferred to microscope slide and illuminated using picosecond laser head. Repetition rate of 485 nm pulse laser was set to 10 Mhz. The fluorescence signal was recorded using single photon detector - PMA Hybrid 40. The photon distribution histogram created by TCSPC electronics based on PicoHarp 300 was then analyzed in Symphotime64 software. Instrument Response Function (IRF) was recorded with fluorescein quenched by saturated potassium iodide.

### Computational Methodology

The theoretical calculations were done using the commercial SCIGRESS program (version FJ 2.7). In the first step the valence, configuration and shape of rings was checked and corrected if necessary. Then the molecule was optimized by performing DFT calculation with the B88-LYP GGA functional and the DZVP basis set. In the same way, the water and the methanol molecules were performed. For the optimized structures several systems were created in which the hydrogen bond between the NOSQ molecule and water molecule or the NOSQ molecule and methanol molecule was marked. For each system, DFT calculations were performed with the same initial conditions as previously.

### Synthesis

**Scheme 1:**
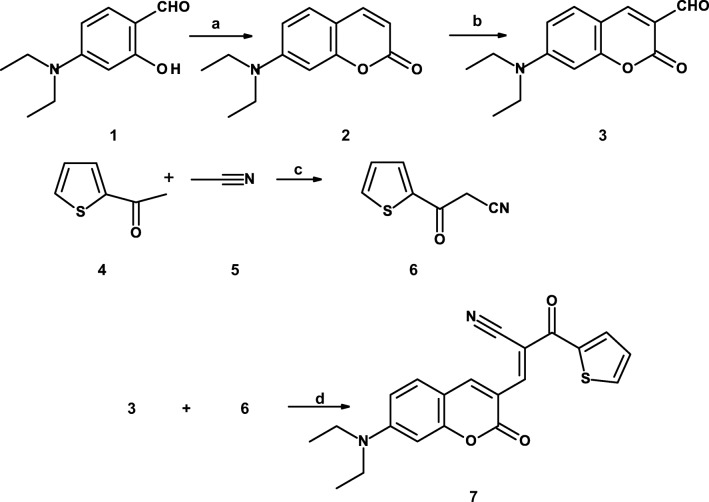
**a** diethyl malonate/piperidine and next HCl; **b** DMF/POCl_3_; **c** toluene/NaH/100 °C/24 h; **d** ethanol/piperidine (cat) /boiling/2 h.

The synthesis of coumarin derivative **7** is summarized in Scheme 1. Compound **3** was synthesized starting from commercially available 4-*N,N*-diethylaminosalicylaldehyde **1** and diethyl malonate. The resulted 7-(*N,N*-diethylamino)coumarin **2** was formylated with DMF/POCl_3_ yielding **3** [20]. The second reagent 3-oxo-3-(2-thienyl)propanenitrile **6** was prepared from 2-acetylthiophene and acetonitrile in the presence of sodium hydride. At last 7-(*N,N*-diethylamino)-3-formylcoumarin **3** and thiophene derivative **6** were reacted together in the presence of catalytic amount of piperidine in boiling ethanol yielding derivative **7**.

A round-bottomed flask (25 mL) equipped with reflux condenser was charged with aldehyde 1 (245 mg, 1 mmol), 3-oxo-3-(2-thienyl)propanenitrile (200 mg, 1.3 mmol), 3 drops of piperidine and ethanol (15 mL). The contents was refluxed for 3 h. After cooling the resulted red precipitate was filtered off and crystallized from toluene. To prepare an analytical sample the resulted compounds was purified on column packed with silica gel Merck 60 (70–230 mesh) using toluene and subsequently toluene/ethyl acetate (3:1) mixture as eluent. Dark crystals with green metallic luster, 273 mg, 72%, mp. 210–11 °C_._^13^C NMR(150 MHz, CDCl_3_): 179.01; 161.11; 158.06; 153.68; 148.61; 144.39; 142.07; 135.04; 133.95; 132.28; 128.45; 118.32; 111.19; 110.45; 108.84; 105.26; 97.20; 45.45; 12.54. ^1^H NMR(300 MHz, CDCl_3_): 8.96(s, 1H); 8.58(1H, s); 7.73(dd, *J* = 4.96 Hz, *J* = 1.01 Hz); 7.44(d, *J* = 9.08 Hz); 7.19–7.18(m, 1H); 6.65(dd, *J* = 9.03 Hz, *J* = 2.48 Hz, 1H); 6.47(d, *J* = 2.19 Hz, 1H); 3.49(q, 4H); 1.26(t, 6H). Anal. calcd for C_21_H_18_N_2_O_3_S: C 66.67%; H 4.79%; N 7.40%; Found C 66.53%; H 4.71%; N 7.28%.

## Results and Discussion

### Calculations

**Fig. 1:**
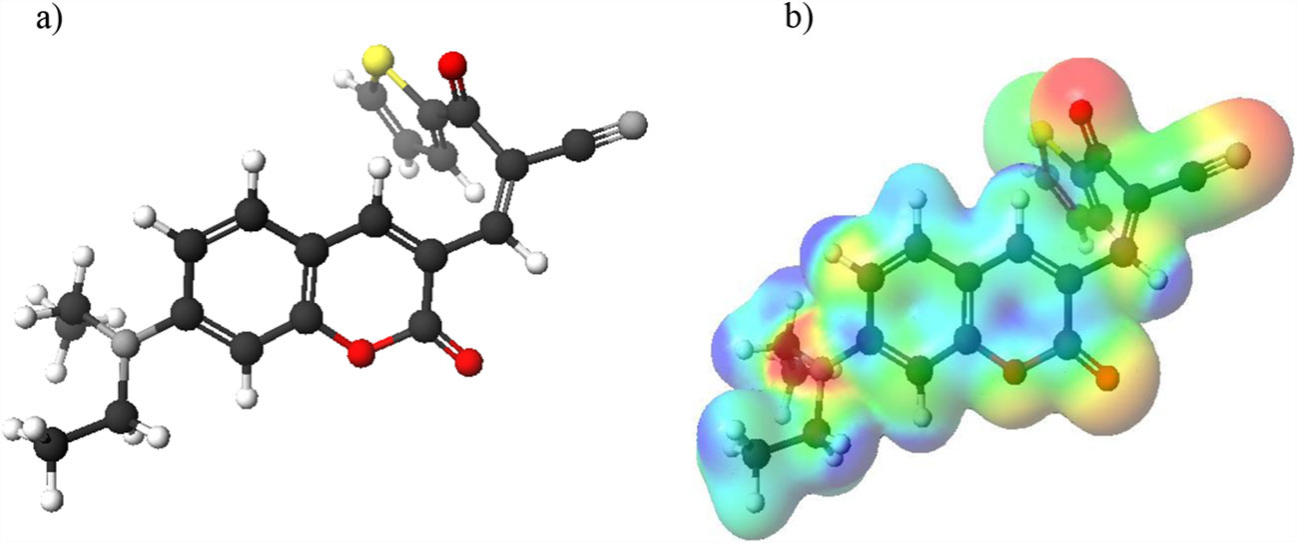
Optimized chemical structure of NOSQ obtained by DFT calculation in Scigress program (**a**). Particular atoms have been marked with colors: carbon – dark grey, hydrogen – white, nitrogen – light grey, oxygen – red and sulfur – yellow. On the right (**b**), the electron density is shown. Red indicates the highest electronegativity and blue indicates positive values of potential

Optimized chemical structure of NOSQ is shown in Fig. 1a. The molecule is polar: the dipole moment in ground state equals 13.4 D. NOSQ molecule has several electronegative atoms with free electron pairs (areas marked in red in Fig. 1b), which can potentially form weak bonds with water or methanol. Taking this into account, pairs of molecules (NOSQ molecule – solvent molecule) were constructed in which a hydrogen bond was formed between the NOSQ molecule and the water molecule or between the NOSQ molecule and the methanol molecule. The further calculations were performed. For obtained NOSQ structures which formed hydrogen bonds with the solvent molecule, the values of dihedral angles in NOSQ molecule were measured and compared with dihedral angles occurring in NOSQ molecule where no weak bonds were considered. The calculated values of dihedral angles for NOSQ molecule without any weak bond and with weak bonds formed with the solvent molecule are collected in Table 2. In most cases the weak bonds do not change the geometry of NOSQ. But there are significant changes in values of dihedral angles when the weak bond is formed between the oxygen atom with double bond attached to the main skeleton of NOSQ molecule and methanol. It shows that there is a possibility of existence of two NOSQ different conformers in water-methanol mixture.

**Table 2:**
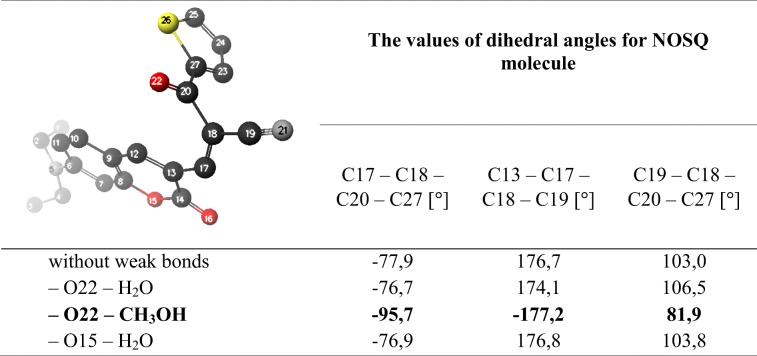
Calculated values of dihedral angles for NOSQ molecule. In the first column, it is pointed out the NOSQ atom that forms a weak bond with the particular solvent.

### Spectral Analysis of NOSQ in Binary Mixture

**Fig. 2:**
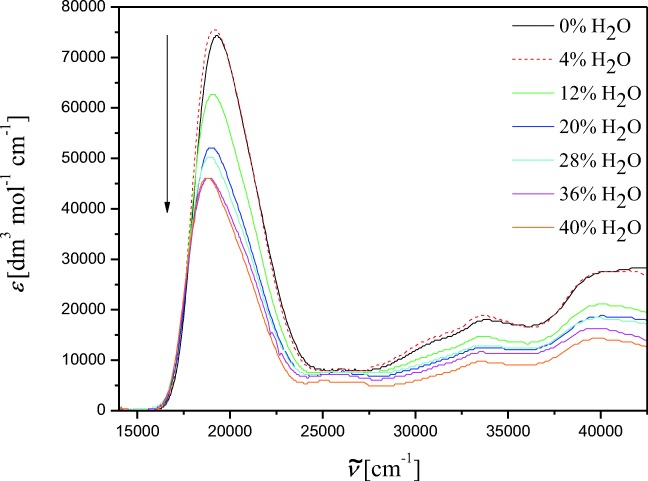
Absorption spectra of NOSQ in binary mixture for different content of water and methanol. For solid lines, the arrow shows the water volume percentage increase in the system

The electronic absorption spectra of NOSQ in pure methanol and water-methanol mixtures are shown in Fig. 2. The most intensive absorption band is observed in the range 16,000–23,000 cm^−1^. The first portion of added water (4% *V*/V) causes slight increase of absorbance value. Next portions of water decrease the absorption intensity. With the increasing of water volumetric fraction from 0% to 40%, the value of absorbance maximum is reduced 2.5 times while the concentration of NOSQ is constant. The red shift with increasing of the percentage of water in the sample is noticed. The dielectric permittivity and refractive index is much bigger for water than for methanol. Therefore the system becomes more polar with increasing water content. From the batochromic shift we conclude that interaction between the molecule and the polar solvent decreases the energy gap between the more polar excited state and the less polar ground one of the molecule.

**Fig. 3:**
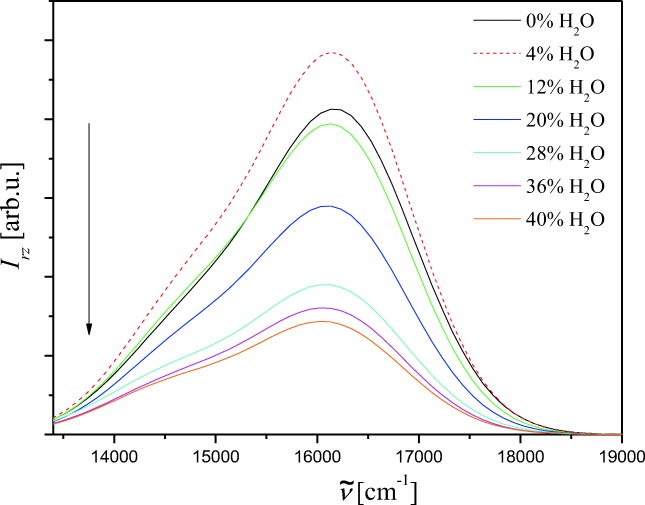
Fluorescence spectra of NOSQ in binary mixture for different content of water and methanol. For solid lines, the arrow shows the water volume percentage increase in the system

The fluorescence spectra are shown in Fig. 3. The dual fluorescence with maxima at 16200 cm^−1^ and 14,500 cm^−1^ is observed. These maxima do not shift with addition of water. The same effect like in absorption is noticed in fluorescence – the band intensity increases after adding the first portion of water and afterwards decreases. The Stokes shift and fluorescence quantum yield are smaller and smaller when the water volumetric percentage increases from 4% to 40% *V*/V. The spectroscopic data for NOSQ in binary mixture, like absorption maximum, the value of the molar absorption coefficient in maximum, Stokes shifts relative to the shortwavelength fluorescence band, fluorescence quantum yield, fluorescence lifetimes and natural fluorescence lifetimes are collected in Table 3. The decay of NOSQ fluorescence in pure methanol and in binary mixture is bi-exponential. This phenomenon and the dual fluorescence suggest the existence of two conformers of NOSQ [21–23]. The experimental results are in good agreement with the theoretical one.

**Table 3:** Photophysical properties of NOSQ in the water-methanol mixture ($\overset{\sim }{\nu }$_*a,*_ [cm^−1^] – absorption maximum, ***ɛ***_***max,abs***_ [dm^3^·mol^−1^·cm^−1^] – the value of the molar absorption coefficient in maximum, ∆$\overset{\sim }{\nu }$ [cm^−1^] – Stokes shifts relative to the short-wavelength fluorescence band, *ϕ*_*f*_ – fluorescence quantum yield, *τ*_1_, *τ*_2_ [ns] – fluorescence lifetime, ${\tau}_1^0$*,*${\tau}_2^0$*–* natural fluorescence lifetime, *f*(*ε,n*) – polarity function)

% of H_2_O	*f*(*ε,n*)	$\overset{\sim }{\nu }$_*a*_	ɛ_*max*, *abs*_	∆ $\overset{\sim }{\nu }$	*ϕ*_*f*_	*τ*_1_	*τ*_2_	${\tau}_1^0$	${\tau}_2^0$
0	13,290	19,300	18,800	2600	0,59	3,51	0,207	5,01	0,298
4	13,292	19,200	19,200	2500	0,87	3,80	0,196	4,37	0,226
8	13,293	19,200	17,200	2500	0,80	3,65	0,186	4,56	0,232
12	13,295	19,100	16,000	2400	0,82	3,70	0,171	4,51	0,208
16	13,296	19,000	15,900	2300	0,74	3,29	0,166	4,45	0,224
20	13,298	19,000	13,400	2300	0,76	4,10	0,166	5,39	0,218
24	13,300	18,900	12,400	2200	0,57	3,43	0,161	6,02	0,282
28	13,301	18,900	13,000	2200	0,52	2,95	0,142	5,67	0,273
32	13,303	18,900	13,000	2200	0,49	3,46	0,142	7,06	0,290
36	13,304	18,800	12,000	2100	0,46	4,24	0,14	9,22	0,304
40	13,306	18,800	10,600	2100	0,48	3,56	0,13	7,42	0,271

**Fig. 4:**
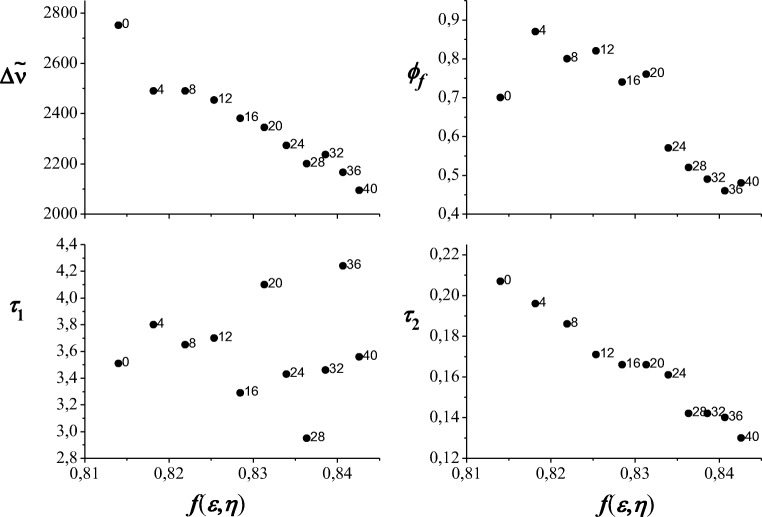
The solvent polarity dependence on Stokes shifts, quantum yield and fluorescence lifetimes

The dependence of solvent polarity (calculated from eq. 3) on Stokes shifts, quantum yield and fluorescence lifetimes are shown on Fig. 4. The quite good linear relation between solvent polarity function and Stokes shifts, quantum yield and shorter fluorescence lifetime, *τ*_2_ is observed. The correlation coefficient is equal 0.97, 0.83 and 0.99 for the *f*(*ε*, *n*) dependence from ∆$\overset{\sim }{\nu }$*, ϕ*_*f*_ and *τ*_2_, respectively. The weakest correlation occurs between polarity function and quantum yield, but at consideration only data of binary mixtures the correlation coefficient increases to 0.95. The first portion of water causes that the quantum yield of NOSQ fluorescence in mixture is higher than in pure methanol. Then this quantum yield decreases, but its values in the water-methanol mixtures are higher than in the pure methanol solution unless the percentage of water is about 25%. Although the fluorescence intensity decreases the quantum yield is high. It can be explained by the smaller and smaller absorbance value at the excitation wavelength when the water is added. No general trend for the polarity dependence of *τ*_1_ is observed but the natural fluorescence lifetime, ${\tau}_1^0$, grows exponentially with increasing polarity of the solutions. The values of Stokes shifts, quantum yield and fluorescence lifetime change predictably when the polarity of the system increases.

## Conclusions

In this paper, we show the synthesis and spectroscopic properties of derivative of coumarin (*E*)-3-[7-(diethyloamino)-2-oxo-chromen-3yl]-2-(tiophene-2-carbonyl)prop-2-enenitrile (NOSQ) in methanol and in water-methanol binary mixtures. The steady state and time-resolved fluorescence indicate the presence of two conformers of NOSQ molecule, which is in agreement with theoretical calculations. The interaction between NOSQ and solvent does not significantly affect molecule geometry, except one case when dihedral angles change. Adding small portions of water (up to about 25% of sample volume) to the methanol solutions is beneficial for the emissive properties of the system. Water is often the quencher, but in this case it increases the quantum yield. This phenomenon can be promising for possibility of application of NOSQ in spectral studies of biological systems, for e.g. as the sensor of ions dissolved in water what is planned in our next studies.
